# Long-term impacts of bush/wildfires on mental health: preparing for the next fire

**DOI:** 10.1192/bjo.2024.737

**Published:** 2024-09-23

**Authors:** Lawrence A. Palinkas

**Affiliations:** Herbert Wertheim School of Public Health and Human Longevity Science, University of California, San Diego, USA

**Keywords:** Climate change, mental health, wildfires, subclinical symptoms, treatment and prevention

## Abstract

Climate change is responsible for marked increases in the frequency and severity of bush/wildfires, resulting in more people exposed to such events with long-term subclinical psychiatric symptoms. This editorial calls for immediate action to implement comprehensive and novel approaches to treating these conditions and preventing them from developing into more severe disorders.

From time immemorial, surviving extreme weather events (EWEs) such as hurricanes, tornadoes, floods and fires has been a fundamental part of human existence. In recent decades, however, our species has had to contend with more frequent and severe EWEs as a consequence of human-induced climate change.^1^ Although some may minimise the significance of these trends and/or continue to deny our responsibility for these events, the evidence of their effects on our mental health is clear and unmistakable.^2^

Among the many forms of EWE associated with changes in our climate has been the bushfire, as it is known in Australia, or the wildfire, as it is known elsewhere. In the past two decades, there has been a proliferation of bush/wildfires (BWFs) that has led to a significant loss of life and property and destruction of the natural environment.^3^ Like other forms of EWE, BWFs are expected to become more frequent and more devastating in the coming decades.^4^

BWFs have been associated with elevated rates of psychiatric disorders, including the ‘trifecta’ of post-traumatic stress disorder, major depressive disorder and generalised anxiety disorder.^5^ Previous research conducted with people exposed to natural disasters suggested that the prevalence of these disorders would diminish over time but remain at rates higher than those of the general population.^6^ However, as illustrated by the study by Pacella and colleagues,^7^ a larger percentage of those exposed to such events may experience subthreshold or subclincial versions of these disorders that may persist for several years. Women, youth, older adults, low-income groups and those with existing mental health problems or living in under-resourced communities are especially vulnerable to these conditions.^2^

It has been well documented that many but not all people experience such mental health problems after a natural disaster, and that such conditions may persist for many but not all of those who experience them. However, what sets this study apart from other post-BWF investigations of mental health impacts is that it demonstrates subclinical symptoms increase the risk of more severe disorders over time. Utilising data from an Australian cohort that had been exposed to the Black Saturday BWF in early 2009, Pacella and colleagues^7^ found that probable adjustment disorder at 3–4 years post-fires predicted a fivefold increase in the risk of escalation to severe psychiatric disorder at 10 years post-fires. The slight increase in prevalence of probable adjustment disorder over time (from 15.8 to 18.6%) suggests that disaster-related subclinical symptoms may remain stable and perhaps even increase, in contrast to the long-held belief in a decline in prevalence and severity of psychological distress directly related to EWE exposure over time.

To account for these findings, the investigators offer evidence that in addition to the acute trauma associated with the immediate threat to life and limb and destruction of property, people exposed to BWFs frequently experience ongoing stressors such as unemployment, housing insecurity, displacement, and disruption of social networks and interpersonal relations. Consistent with Hobfoll's conservation of resources theory,^8^ these ongoing stressors maintain high levels of psychological distress which, in turn, increase the risk of exposure to these stressors.

BWFs also damage the environment in ways that jeopardise mental health. Exposure to BWFs and other EWEs can trigger ‘psychoterratic’ (i.e. earth-related) syndromes such as solastalgia (distress produced by environmental change when directly connected to one's home environment), eco-anxiety (the chronic fear of environmental cataclysm that comes from observing the seemingly irrevocable impact of climate change) and eco-paralysis (the inability to meaningfully respond to climatic and ecological challenges).^9^ These syndromes, in turn, can lead to clinically significant psychological distress.^10^ Climate anxiety is especially prominent among children and young people,^9^ making them vulnerable to post-BWE subthreshold disorders. A survey of 10 000 children and young people (aged 16–25 years) in ten countries found that 59% were very or extremely worried and 84% were at least moderately worried about climate change.^11^

Gas-phase chemicals and particulate matter emitted into the atmosphere by BWFs have also been linked to increased risk of depression, suicide and other mental health problems.^12^ PM_2.5_ (particulate matter where particles are less than 2.5 micrometres in diameter), in particular, has been found to overcome the blood–brain barrier through extrapulmonary or olfactory routes and cause direct inflammation and oxidative stress in the brain. This inflammation has been hypothesised to trigger difficulties with cognitive functioning and the development of mood symptoms.^13^

The study by Pacella et al also points to the need to implement novel interventions to mitigate subclinical symptoms, which themselves are debilitating, and prevent their escalation to more severe psychiatric disorders. Individuals experiencing subclinical symptoms after a BWF event may not require the intensive and expensive treatments that are recommended for those with more severe psychiatric disorders. Rather, approaches such as the use of low-intensity interventions such as Problem Management Plus,^14^ Skills for Psychological Recovery^15^ and Skills for Life Adjustment and Resilience^16^ may be more appropriate and cost-effective in treating people exposed to BWFs.^17^ Such interventions can be delivered by non-mental-health professionals such as community health workers, which is particularly beneficial in rural areas and low-income countries where the availability of trained mental health professionals is limited. There is also promising evidence that non-mental-health professionals can be trained to provide low intensity trauma-informed interventions such as Support for Students Exposed to Trauma for school-aged youth.^18^ These interventions have been found to be effective in treating post-traumatic stress, depression and anxiety in children and adolescents exposed to EWEs and other traumatic events. Research has also provided evidence of the effectiveness of web-based and mobile health (i.e. mHealth) interventions for certain population subgroups such as adolescents recovering from BWFs and other natural disasters.^19,20^

Based on their own findings, Pacella and colleagues recommend long-term practical support to target post-disaster stressors such as loss of income due to unemployment, food and housing insecurity, and dysfunctional interpersonal relationships. A comprehensive approach to the prevention and treatment of subclinical symptoms and more severe disorders will require coordinated planning and preparedness that includes inventories of existing resources and identification of at-risk populations, training of teachers and community health workers to deliver mental health services to these at-risk populations, routine and ongoing screening and assessment of people exposed to BWFs and other forms of EWE, and development and implementation of effective and sustainable programmes to strengthen individual and community resilience.

The paucity of research in general and the absence of controls or accounting for attrition bias in the Pacella et al study, which may affect the resulting prevalence and risk estimates, highlight a need for further research on these phenomena and their significance. Moreover, BWF is only one of several different types of EWE believed to be influenced by climate change. Nevertheless, the long-term subclinical symptoms observed in the aftermath of the Black Saturday BWF in Australia are likely to be experienced by increasing numbers of people exposed to other forms of EWE with greater intensity and duration in the future. Furthermore, the problem will only grow worse with repeated exposure to such events as predicted by current climate models.^1^ Climate change represents one of the most significant mental health crises of our time, if not the most significant, with profound implications for our lives and the lives of future generations. Although humankind has experienced natural disasters throughout its history, a new approach to protecting our mental health in the aftermath of such events is necessary. Symptoms that might have been ignored in the past because they failed to meet DSM or ICD diagnostic criteria or were assumed to diminish over time must be the central focus of this new approach. We cannot afford to wait for the next fire.

## Data Availability

Data availability is not applicable to this article as no new data were created or analysed in this work.

## References

[1] Intergovernmental Panel on Climate Change. Climate Change 2022: Impacts, Adaptation and Vulnerability. Contribution of Working Group II to the Sixth Assessment Report of the Intergovernmental Panel on Climate Change. Cambridge University Press, 2022 (https://www.ipcc.ch/report/ar6/wg2/ [cited 12 Feb. 2024]).

[2] Palinkas LA, Wong M. Global climate change and mental health. Curr Opin Psychol 2020; 32: 12–6. 31349129 10.1016/j.copsyc.2019.06.023

[3] Abatzoglou JT, Williams AP. Impact of anthropogenic climate change on wildfire across western US forests. Proc Natl Acad Sci USA 2016; 113: 11770–5. 27791053 10.1073/pnas.1607171113 PMC5081637

[4] Goss M, Swain DL, Abatzoglou JT, Sarhadi A, Kolden CA, Williams AP, et al. Climate change is increasing the likelihood of extreme autumn wildfire conditions across California. Environ Res Lett 2020; 15: 094016.

[5] Bryant R, Waters E, Gibbs L, Gallagher HC, Pattison P, Lusher D, et al. Psychological outcomes following the Victorian Black Saturday bushfires. Aust N Z J Psychiatry 2014; 48: 634–43. 24852323 10.1177/0004867414534476

[6] Norris FH, Friedman MJ, Watson PJ, Byrne CM, Diaz E, Kaniasty K. 60,000 disaster victims speak: part 1. An empirical review of the empirical literature, 1981–2001. Psychiatry 2002; 65: 207–39. 12405079 10.1521/psyc.65.3.207.20173

[7] Pacella B, Cowlishaw S, Gibbs L, Bryant R, Gallgher C, Molyneaux R, et al. Trajectory of adjustment difficulties following disaster: 10-year longitudinal cohort study. Br J Psychiatry 2024; 10(2): e57. 10.1192/bjo.2024.3 PMC10951843 38433588

[8] Hobfoll S. Conservation of resources and disaster in cultural context: the caravans and passageways for resources. Psychiatry 2012; 75(3): 227–32. 22913498 10.1521/psyc.2012.75.3.227

[9] Clayton S. Climate anxiety: psychological response to climate change. J Affect Disord 2020; 74: 102263. 10.1016/j.janxdis.2020.102263 32623280

[10] Eisenman D, McCaffrey S, Donatello I, Marshal G. An ecosystems and vulnerable populations perspective on solastalgia and psychological distress after a wildfire. Ecohealth 2015; 12(4): 602–10. 26302957 10.1007/s10393-015-1052-1

[11] Hickman C, Marks E, Pihkala P, Clayton S, Lewandowski RE, Mayall EE, et al. Climate anxiety in children and young people and their beliefs about government responses to climate change: a global survey. Lancet Planetary Health 2021; 5(12): e363–73. 10.1016/S2542-5196(21)00278-3 34895496

[12] Eisenman DP, Galway LP. The mental health and well-being effects of wildfire smoke: a scoping review. BMC Public Health 2022; 22(1): 2274. 36471306 10.1186/s12889-022-14662-z PMC9724257

[13] Buoli M, Grassi S, Caldiroli A, Carnevali GS, Mucci F, Iodice S, et al. Is there a link between air pollution and mental disorders? Environ Int 2018; 118: 154–68. 29883762 10.1016/j.envint.2018.05.044

[14] Dawson KS, Bryant RA, Harper M, Tay AK, Rahman A, Schafer A, et al. Problem Management Plus (PM+): a WHO transdiagnostic psychological intervention for common mental health problems. World Psychiatry 2015; 14: 354–7. 26407793 10.1002/wps.20255 PMC4592660

[15] Heinz AJ, Wiltsey-Stirman S, Sharin T, Loskot T, Mason D, Jaworski BK, et al. Rising from the ashes by expanding access to community care after disaster: an origin story of the wildfire mental health collaborative and preliminary findings. Psychol Serv 2022; 19(Suppl 2): 58–66. 10.1037/ser0000553 34180706

[16] O'Donnell ML, Lau W, Fredrickson J, Gibson K, Bryant RA, Bisson J, et al. An open label pilot study of a brief psychosocial intervention for disaster and trauma survivors. Front Psychiatry 2020; 11: 483. 32670099 10.3389/fpsyt.2020.00483 PMC7332836

[17] Massazza A, Eaton J, Elshazly M, Charlson F, Augustinavicius JL. Opportunities for the use of brief scalable psychological interventions to support mental health and well-being in the context of the climate crisis. Intervention 2022; 20(1): 128–35.

[18] Jaycox LH, Langley AK, Stein BD, Wong M, Sharma P, Scott M, et al. Support for students exposed to trauma: a pilot study. School Mental Health 2009; 1(2): 49–60. 20811511 10.1007/s12310-009-9007-8 PMC2930829

[19] Ruggiero KJ, Price M, Adams Z, Stauffacher KM, McCauley J, Danielson CK, et al. Web intervention for adolescents affected by disaster: population-based randomized controlled trial. J Acad Child Adolesc Psychiatry 2015; 54: 709–17. 10.1016/j.jaac.2015.07.001 PMC4548271 26299292

[20] Obuobi-Donkor G, Shalaby R, Agyapong B, Dias RDL, Agyapong VIO. Mitigating psychological problems associated with the 2023 wildfires in Alberta and Nova Scotia: six-week outcomes from the Text4Hope program. J Clin Med 2024; 13(3): 865. 38337558 10.3390/jcm13030865 PMC10856019

